# Supplementary material for: Discovery of a small molecule that inhibits bacterial ribosome biogenesis
Source: eLife. 2014 Sep 18;3:e03574. doi: 10.7554/eLife.03574 (PMC4371806; doi:10.7554/eLife.03574)
Supplement: Supplementary file 2. — Related to Figure 5. Temperature-dependent activity of lamotrigine across bacterial species. DOI: http://dx.doi.org/10.7554/eLife.03574.023 [file elife03574s006.docx]

|  | **37°C MIC** | **15°C MIC** | **Fold ΔMIC** |
| --- | --- | --- | --- |
| *Enterococcus faecalis* | >500μM | >500μM |  |
| ***Escherichia coli*** | **>500μM** | **7.8μM** | **>64** |
| ***Citrobacter rodentium*** | **>500μM** | **15.6μM** | **>32** |
| ***Salmonella enterica*** | **>500μM** | **15.6μM** | **>32** |
| ***Enterobacter cancerogenus*** | **>500μM** | **62.5μM** | **>8** |
| *Pseudomonas aeruginosa* | >500μM | >500μM |  |
| *Acinetobacter baumanni* | >500μM | >500μM |  |

**Supplementary File 2 – related to Figure 5.** Temperature-dependent activity of lamotrigine across bacterial species.
